# A roadmap for translational cancer glycoimmunology at single cell resolution

**DOI:** 10.1186/s13046-022-02335-z

**Published:** 2022-04-15

**Authors:** Andreia Peixoto, Andreia Miranda, Lúcio Lara Santos, José Alexandre Ferreira

**Affiliations:** 1 Experimental Pathology and Therapeutics Group, IPO Porto Research Center (CI-IPOP), Portuguese Oncology Institute (IPO Porto), 4200-072 Porto, Portugal; 2 Institute for Research and Innovation in Health (i3S), University of Porto, 4200-135 Porto, Portugal; 3 Institute for Biomedical Engineering (INEB), University of Porto, 4200-135 Porto, Portugal; 4 Porto Comprehensive Cancer Center (P.Ccc), 4200-072 Porto, Portugal; 5 Faculty of Medicine, Porto University (FMUP), 4200-319 Porto, Portugal; 6 Health School of University Fernando Pessoa, 4249-004 Porto, Portugal; 7 GlycoMatters Biotech, 4500-162 Espinho, Portugal; 8 Department of Surgical Oncology, Portuguese Oncology Institute (IPO Porto), 4200-072 Porto, Portugal; 9 Institute of Biomedical Sciences Abel Salazar (ICBAS), University of Porto, 4050-013 Porto, Portugal

**Keywords:** Cancer glycosylation, Glycoimmunology, Cancer models, Cancer, Translational research

## Abstract

Cancer cells can evade immune responses by exploiting inhibitory immune checkpoints. Immune checkpoint inhibitor (ICI) therapies based on anti-CTLA-4 and anti-PD-1/PD-L1 antibodies have been extensively explored over the recent years to unleash otherwise compromised anti-cancer immune responses. However, it is also well established that immune suppression is a multifactorial process involving an intricate crosstalk between cancer cells and the immune systems. The cancer glycome is emerging as a relevant source of immune checkpoints governing immunosuppressive behaviour in immune cells, paving an avenue for novel immunotherapeutic options. This review addresses the current state-of-the-art concerning the role played by glycans controlling innate and adaptive immune responses, while shedding light on available experimental models for glycoimmunology. We also emphasize the tremendous progress observed in the development of humanized models for immunology, the paramount contribution of advances in high-throughput single-cell analysis in this context, and the importance of including predictive machine learning algorithms in translational research. This may constitute an important roadmap for glycoimmunology, supporting careful adoption of models foreseeing clinical translation of fundamental glycobiology knowledge towards next generation immunotherapies.

## Introduction

**Fig. 1:**
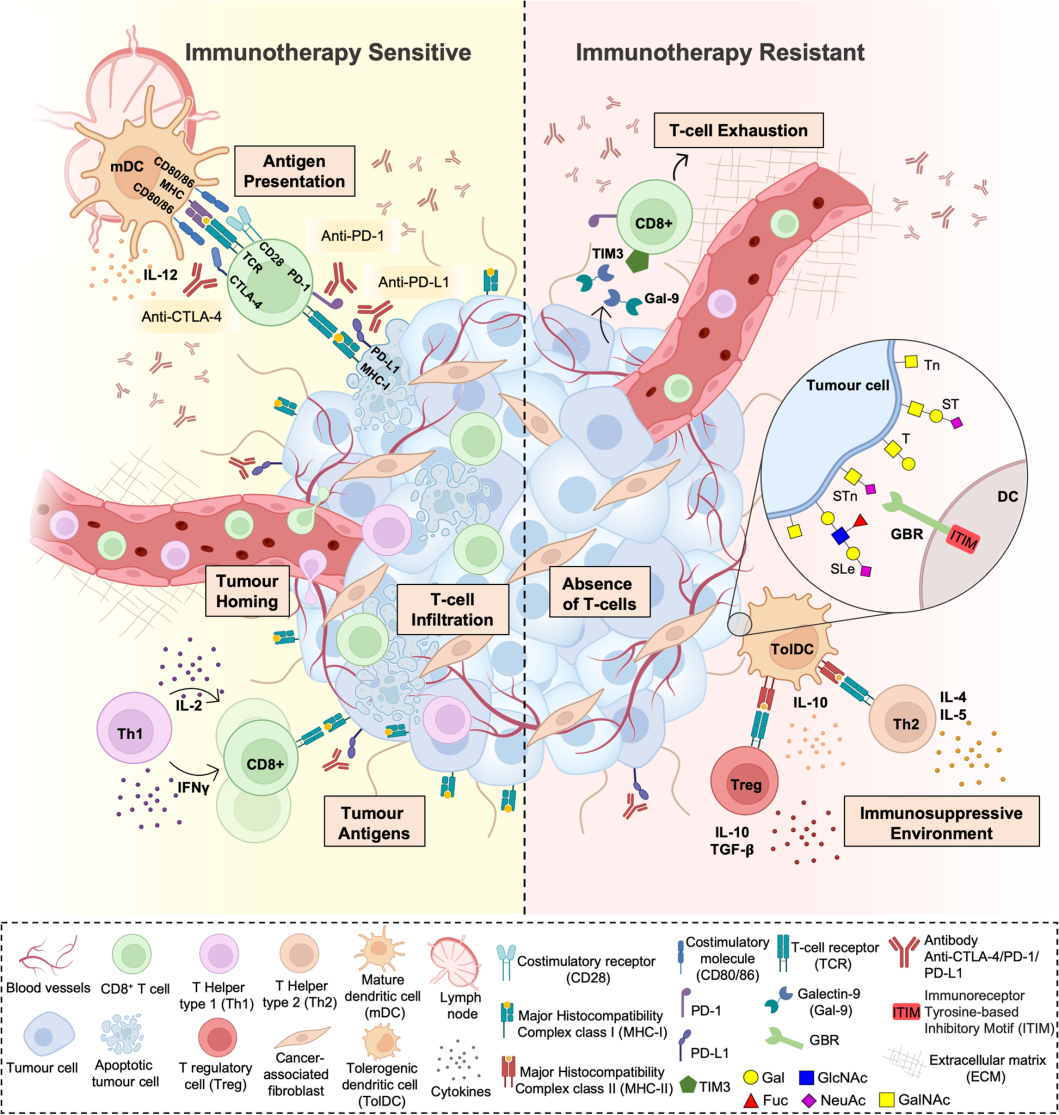
Currently accepted immune checkpoint inhibitors resistance mechanisms**.** Resistance to currently used immune checkpoint inhibitors, namely anti-PD1, anti-PD-L1 and anti-CTLA-4 therapeutic antibodies, has been associated with the lack of effective tumour T cell infiltration. This could be due to lack of tumour antigens, defects in antigen presentation, and absence of T cell activation. Of note, cancer-associated glycosignatures, (e.g. Tn, STn, lewis antigens, among others) that are distinct from those found on healthy cells, can interact with glycan-binding receptors (GBR) in immune cells, driving immunosuppression. Glycan-mediated immunosuppression is promoted by non-classical pathways, including altered antigen uptake, processing, and presentation by antigen presenting cells, ultimately conditioning T-cell priming. Moreover, GBRs engagement alters immune cell signalling, differentiation, and cytokine responses toward anti-inflammatory or immunosuppressive phenotypes. Furthermore, the abnormal glycosylation of tumour-associated glycoproteins generates neoantigens that can serve as targets for tumour-specific T cells

Over the last decade, the introduction of T cell targeted immunomodulators blocking immune checkpoints CTLA-4 and PD1 or PDL1 has been unprecedented, with immune checkpoint inhibitors (ICI) being used as single agents or in combination with chemotherapies in about 50 cancer types [1]. Despite its efficiency in high mutational load tumours, such as melanoma and lung cancer [2, 3], ICI does not meet its promise for most cancer patients. The overall response rate to ICI is approximately 12% in low mutational burden tumours, which increases the demand for biomarkers of response and alternative therapeutic strategies to overcome therapy resistance [4]. Furthermore, patients non-responding to immune checkpoint inhibitors experience a vast panoply of immune-mediated iatrogenic effects [5], leading to treatment discontinuation, or become refractory [6]. Initial resistance to ICI has been associated with the lack of T cell infiltration (cold tumour phenotype) (Fig. 1). Recently, the main mechanisms governing poor T cell infiltration in cold tumours were described, including lack of tumour antigens, defects in antigen presentation, absence of T cell activation and deficit of immune homing into the tumour bed [7], all of these factors being highly dependent on the tumour microenvironment (TME) [8] and likely enhanced by aberrant tumour glycosylation [9] (Fig. 1).

**Fig. 2:**
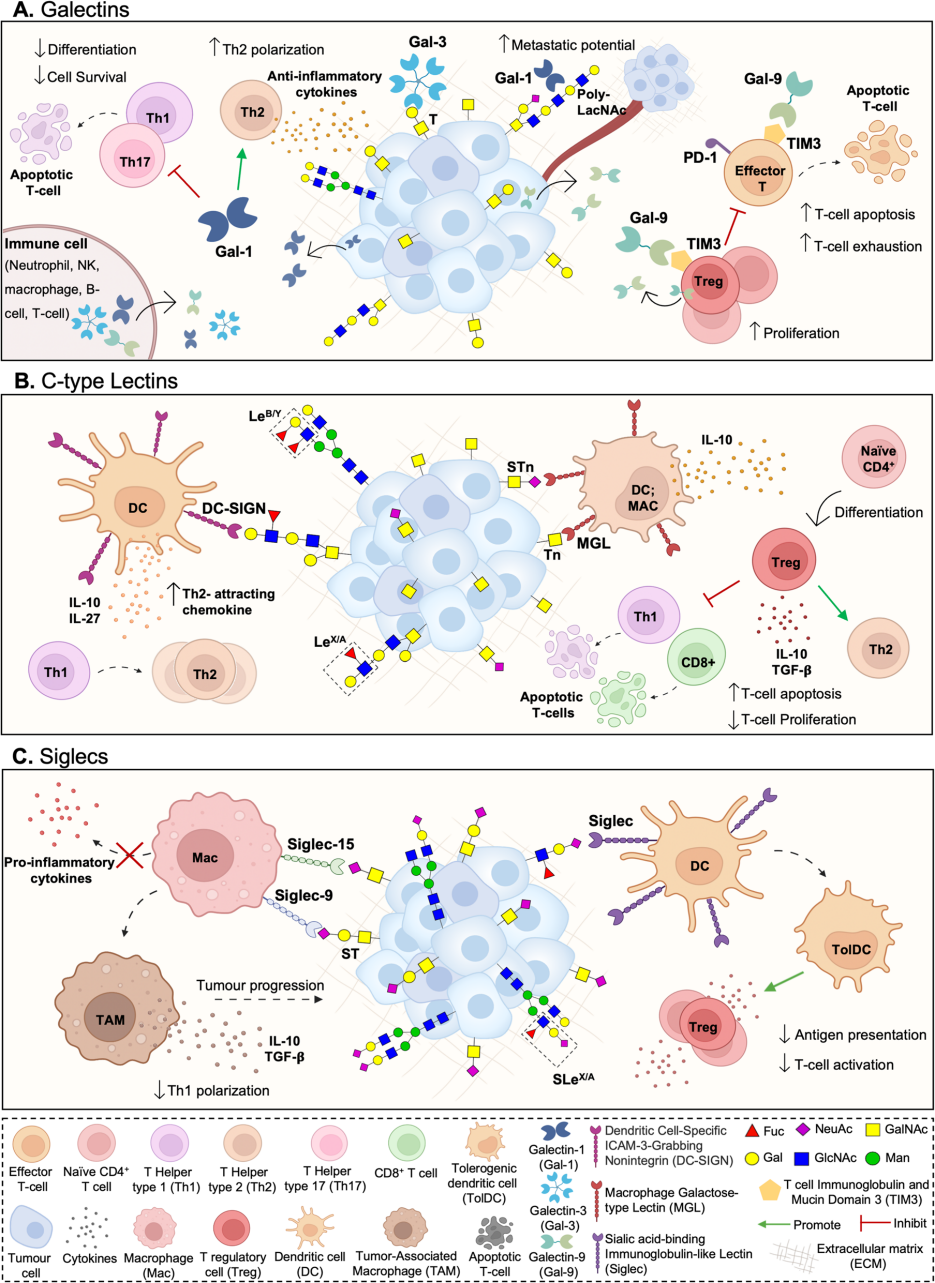
Glycan binding receptors (GBR) interactions with tumour cell aberrant glycosylation as a novel class of immune checkpoints**.** Cancer cell associated glycans modulate immune cell responses by interacting with various classes of GBR, as galectins (A), most C-type lectins (B) and siglecs (C). Galectins (Gal) are secreted lectins that bind terminal β-galactoside-containing carbohydrates, including the T antigen. Tumour secreted galectins can induce apoptosis of activated human T cells, while antagonizing Type 1 T helper (Th1) and Type 17 T helper (Th17) cells survival. Furthermore, exhausted T cells frequently express galectin ligands, as the T cell immunoglobulin and mucin domain 3 (TIM3), regulating T cell exhaustion while modulating the inhibitory action of CD4 + CD25 + regulatory T cells (Tregs). Furthermore, galectins endow dendritic cells (DCs) with a tolerogenic phenotype capable of promoting interleukin 10 (IL-10)-mediated T cell tolerance. In turn, C-type lectin-like domain superfamily members as DC-SIGN, that avidly recognizes fucosylated lewis antigens on *O*- and *N*-glycans, and MGL, with affinity for terminal GalNAc (Tn antigen), are widely expressed by DCs and macrophages. Particularly, DC-SIGN engagement in the tumour microenvironment enhances interleukin -10 and -27, as well as Th2-attracting chemokine expression, shifting Thelper polarization from Th1 to Th2. MGL interaction with Tn antigens, and possibly STn, is also known to partially abrogate Th1 cell responses by promoting IL-17 and IL-10 expression, reducing effector T cell proliferation, and inducing T cell apoptosis. Siglecs are sialic acid-binding immunoglobulin-type lectins expressed by immune cells which selectively recognize tumour cell sialic acids, including STn and ST antigens. By interacting with tumour associated sialoglycans, siglecs modulate tolerogenic functions in DCs, preventing expansion of effector CD4 + and CD8 + T cells and increasing Treg cell numbers. In particular, STn and ST antigens seem to be potent agonists of inhibitory siglecs, as macrophage Siglec-15 and -9, respectively, inducing tumour-associated macrophage (TAM)-like phenotypes and permitting immune scape

Over 40 years of cancer glycobiology studies support the notion that the glycome at the cell-surface of cancer cells, extracellular vesicles and secreted/released molecules is fundamentally altered, generating cancer-associated glycosignatures that are distinct from those of healthy cells [10–14]. However, while their contribution to cancer aggressiveness has been subject of much interest, the interplay with the immune system has remained mostly overlooked. Nevertheless, it is rather consensual that alterations in glycosylation reshape tumour recognition by the immune system and induce immunosuppressive signalling through glycan-binding receptors (GBR) [15] (Fig. 1). Namely, altered glycosylation directly impacts on antigen presentation by modulating antigen presenting cells (APC) uptake of abnormally glycosylated proteins, its proteolytic processing and presentation by Major Histocompatibility Complexes (MHC), ultimately governing subsequent T-cell priming [16]. Moreover, glycan interactions with GBRs, namely galectins, C-type lectins, and siglecs on APCs is known to alter immune cell signalling, differentiation, and cytokine responses toward anti-inflammatory or immunosuppressive phenotypes [9, 15, 17] (Fig. 2). Furthermore, the abnormal glycosylation of tumour-associated glycoproteins generates neoantigens that can serve as targets for tumour-specific T cells [15]. Collectively, these findings support the relevance of engaging in a thorough understanding of the glycan-immune system interplay towards next generation immunotherapies. The election of appropriate models is critical to fully disclose this intricate crosstalk as well as support therapeutic design and pre-clinical trials. As such, the present review addresses recent studies on this field, emphasizing advantages and disadvantages of available and emerging pre-clinical models in the road towards translational glycoimmunology.

## Cancer associated glycosylation

Aberrant glycosylation is a prominent feature of advanced stage solid tumours [14, 18–20]. These changes are mainly driven by altered expression/activity of glycosyltransferases and glycosidases [21], mislocalization of glycosyltransferases throughout the protein secretory pathways [22], epigenetic silencing of molecular chaperones, as *COSMC* [23], and variations in the bioavailability of sugar donors [21]. Several tumour microenvironmental factors have been suggested to be upstream of glycan-associated phenotypic changes, including variations in oxygen [11], nutrients [24], and inflammatory cytokines levels [25]. The underlying structural alterations contribute to all accepted cancer hallmarks [9], highlighting the critical functional implications of glycans in cancer cells behaviour and fate [11, 26, 27], with considerable negative impact in clinical outcomes [10, 28, 29]. The most well-known glycome alterations result from premature stop in protein *O*-GalNAc glycosylation (occurring in Ser/Thr residues of proteins), yielding immature glycans, such as the Tn, sialyl-Tn (STn) and T antigens, rather than more extended and complex glycosidic chains. The functional and clinical implication of such glycoepitopes has been extensively revised by us and other authors [9, 14, 29, 30]. In brief, the simplest *O*-GalNAc glycan, the Tn antigen, is expressed by 10–90% of human epithelial tumours, including bladder, ovary, lung, breast, cervix, colon, stomach, and prostate cancer, while being mostly absent form healthy tissues [31–33]. In cancer, Tn antigen expression has been correlated to poor prognosis and metastasis [34]; however the molecular mechanisms through which it modulates tumour progression remains poorly understood and appears to be dependent on the microenvironment, requiring comprehensive systems biology approaches for elucidation. Notwithstanding, Tn antigen expression seems to significantly impact the tumour-associated immune cell repertoire, which translates in reduced levels of cytotoxic CD8 + T cells and enhanced accumulation of myeloid-derived suppressor cells [35, 36]. Furthermore, Tn antigen is involved in the adhesion of tumour cells to the endothelium via a mechanism recruiting Galectin-3 and MUC-1, which is one of the first steps of metastasis formation [34]. This processes ultimately accelerate tumour growth in vivo, providing pathways for therapeutical intervention. Based on these observations, several therapeutic approaches against Tn antigen are in pre-clinical development or in clinical trials, including glycovaccines [37–39], cellular immunotherapies, as chimeric antigen receptor T cells (CAR-T) [40, 41], and monoclonal antibodies [38, 42]. The sialylated Tn antigen, or sialyl-Tn (STn), is also frequently expressed in tumour tissues, including breast, gastrointestinal, lung, prostate, oesophagus, and bladder cancers [43]. It modulates key aspects of cancer progression, being an independent predictor of poor prognosis [14, 29, 43] frequently found in glycoproteins intimately linked to cancer aggressiveness [10, 27]. Numerous reports describe STn as a driver of decreased cell adhesion [11, 44], increased migratory and invasive [11, 45] capacity of tumour cells, and decreased chemotherapy-induced apoptosis [46]. Its presence in highly undifferentiated circulating tumour cells and metastases also suggests a potential role in metastasis [28]. Finally, STn is a potent inducer of cancer tolerogenecity in immune cells, impairing both dendritic cell maturation and anti-tumour T cell responses [47], while inducing multipotent growth factor production by tumour associated macrophages [48]. Given such tremendous body of evidence supporting STn as a key modulator of tumour growth and progression, several STn targeted therapeutics are in pre-clinical stage of development, including humanized antibodies [49–52], glycovaccines [53, 54] and glycomimetics for immunization [55, 56]. In turn, the core 1 *O*-glycan T antigen is also expressed by 90% of human carcinomas, including precancerous lesions [57] and disseminated tumour cells [58]. In cancer, T antigen serves as a specific ligand of galectin-3, providing a pivotal interaction for metastatic cell adhesion to endothelial cells in several cancer models [59–62]. Moreover, T antigen overexpression was associated with increased invasive capacity and stem-like properties of cancer cells [63], as well as with cancer cells metastatic potential irrespectively of galectin-3 assessment [64]. In line with this, targeted pre-clinical therapeutics against T antigen include clinical immunolocalization antibodies [65], humanized antibodies for immunotherapy [66], and cancer vaccines [67, 68]. Sialylated T antigens (ST) are also a common post-translational modification of membrane glycoproteins. Despite its non-cancer specific nature, it has been found overexpressed in numerous cancers in comparison to healthy tissues [69]. Namely, overexpression of *ST3Gal-I*, responsible for ST biosynthesis, is a biomarker of metastatic potential [70], while ST antigen was demonstrated to impact on tumour cell proliferation, migration, and apoptosis. Of note, ST antigen has also been identified in several key cancer glycoproteins, some of which displaying prognostic potential [27, 69].

Polylactosamine structures present on tri and tetra-antennary *N*-glycans are also commonly found in cancer, especially as result of *β*1–6 Mannose branching, whose synthesis is under control of *GlcNAcT-V* [71]. This originates functionally diverse *N*-glycosidic chains in relevant cancer-associated proteins that are profoundly involved in cancer growth, invasion, metastasis, and immune recognition [68, 71–73], while being correlated with poor prognosis [74]. The negative functional impact of this class of *N*-glycans has been thereby targeted in pre-clinical studies exploiting glycosyltransferases silencing towards potentiated immune recognition [73] and cell based immunotherapies [75]. Polylactosamines are further modified by the addition of different carbohydrate antigens such as Lewis antigens and their sialylated counterparts. Accordingly, another typical glycome-related alteration in cancer cells is the overexpression of sialyl lewis antigens, namely sialyl lewis A (SLe^A^) and sialyl lewis X (SLe^X^). These are terminal epitopes of extended *N*- and *O*-glycosidic chains of glycoproteins and glycolipids. Due to their affinity for selectins expressed on endothelial cells (E-selectin), platelets (P-selectin) and leukocytes (L-selectin), these glycans are key mediators of cancer cells recruitment to activated endothelial cells in primary tumour sites, intravasation into the blood stream and homing to distant locations. Moreover, their circulating levels are frequently used in the clinics as disease monitoring tools [14, 76, 77]. Besides adhesion, SLe antigens also influence angiogenesis [78–80] and immune recognition of cancer cells [81, 82]. For instance, SLe-P-selectin interactions contribute to protect circulating tumour cells against shear forces and immune recognition by forming shielding platelet cloaks [83]. This interaction also contributes to tumour cell adhesion to metastasis sites, as recently reviewed [83]. Given this rational, several therapeutic tools have been developed against such structures, including therapeutic antibodies [84, 85], gene therapy [86], and CAR T-cell immunotherapy [87].

These findings reinforce the remarkable contribution of the glycome to disease progression and dissemination in the context of well-known cancer hallmarks [9]. Furthermore, several recent studies have put glycans in the spotlight as novel checkpoints in compromised immune responses that should be accounted for in terms of patient stratification and therapeutic development. This rationale has also served as basis for glycoproteomics studies, supported by dedicated and progressively more standardized mass spectrometry workflows [88], which have now started to unveil the nature of the proteins carrying many of the described post-translational modifications. Blueprints regarding the existence of cancer unique and context-dependent glycoproteomes have already been provided [13, 27, 89], paving the way towards precise cancer targeting and personalization. These advances will be of major importance for decoding the crosstalk between cancer cells and the immune system towards clinical intervention. However, it becomes pressing that this effort is followed by the election and refinement of cellular and animal models for translational clinical research, crucial for both functional studies and development of glycan-based immunotherapies. Therefore, subsequent sections will explore and summarize glycan mediated immune modulation of innate and adaptative responses known to date, while shedding light on the potentialities and hurdles associated to the most used disease models.

## Immune modulation promoted by cancer-associated glycans

The influence of cell surface glycosylation on innate and adaptive immune responses has been consistently explored in the context of inflammation, autoimmunity and cancer, opening an avenue for the new field of glycoimmunology [15, 90]. More recently, several tumour associated glycans have been suggested as novel immune checkpoint intermediates [15], as their negative impact on immune cell differentiation and antigen-presenting cell functions [91] starts to be unveiled. Briefly, cancer cell associated glycans modulate immune cell responses by interacting with various classes of secreted or membrane-bound glycan-binding receptors, as galectins, most C-type lectins and siglecs [92–94] (Fig. 2). The carbohydrate specificity of these receptors and their immune cell subpopulation biodistribution is well documented and summarized elsewhere [15, 90]. Herein, we will focus on the glycan binding receptor-glycan interactions in the control of innate and adaptive immune responses. Briefly, galectins (Gal) are soluble lectins that are secreted via non-classical pathways to interact with cell surface glycoproteins and extracellular matrix (ECM) ligands [95]. Galectins typically bind terminal β-galactoside-containing carbohydrates with high specificity [96], including the short core 1 *O*-glycan T antigen and polylactosamine structures present on tri and tetra-antennary *N*-glycans (Fig. 2). Notwithstanding, the glycan-binding specificity of each galectin is governed by sulfation, sialylation, fucosylation, repeating *N*-acetyllactosamine units and *β*1,6 GlcNAc branching of the glycan moiety, differing between individual family members [97]. Found in a wide variety of immune cells, galectins as Gal-1 can induce apoptosis of activated human T cells [98], while antagonizing Type 1 T helper (Th1) and Type 17 T helper (Th17) cells survival [99]. Promotion of T-cell receptor (TCR)-induced Type 2 T helper (Th2) cytokine production [100] towards immunosuppressive microenvironments has also been described. Galectin ligands, as the T cell immunoglobulin and mucin domain 3 (TIM3), can be found in exhausted T cells concomitantly expressing PD-1, negatively regulating T cell exhaustion and immunotherapy efficacy in a glycan dependent manner [101, 102] (Fig. 2). On the same note, the TIM3-Gal-9 pathway has consistently been implicated in the inhibitory action of CD4 + CD25 + regulatory T cells (Tregs), reinforcing its immunosuppressive role [103]. Furthermore, galectins can endow dendritic cells (DCs) with a tolerogenic phenotype capable of promoting interleukin 10 (IL-10)-mediated T cell tolerance [104]. On the same note, galectins expressed by innate immune cells, such as polymorphonuclear neutrophils [105], macrophages [106], NK cells [107, 108], and B cells [109, 110] can also interact with glycoconjugates at the surface of tumour cells and ultimately generate a local microenvironment that is permissive to tumour growth.

In turn, the C-type lectin-like domain superfamily is a calcium depend receptor class that often comprises carbohydrate recognition domains [111]. Depending on glycan recognition and calcium coordination [112], C-type lectin receptors (CLRs) are classified as group I mannose-specific (with affinity to terminal mannose and fucose) or group II galactose-specific (with affinity to terminal galactose and *N*-acetylgalactosamine) [94]. In particular, group II receptors are important pattern recognition receptors expressed by APCs, such as DCs and macrophages [94]. These include, the myeloid DC receptor DC-SIGN (CD209) that avidly recognizes fucosylated Lewis antigen structures, and MGL (CLEC10A, CD301), with affinity for terminal GalNAc (Tn antigen), among others [94] (Fig. 2). As previously mentioned, malignant transformation is often accompanied by overexpression of these receptors’ ligands, tailoring suppressive immune responses on tumour sites. Particularly, DC-SIGN engagement in the tumour microenvironment by fucose-based pathogen-associated molecular patterns (PAMPs) enhances interleukin -10 and -27 (IL-10, IL-27), as well as Th2-attracting chemokine expression, shifting Thelper polarization from Th1 to Th2 [113–115]. Reduction of T cell proliferation [116] has also been described upon DC-SIGN engagement. In turn, MGL interaction with shortened glycan moieties, as Tn antigens (GalNAc), strongly affects B and T cell immunogenicity, partially abrogating Th1 cell responses, promoting IL-17 and IL-10 expression [117, 118], reducing effector T cell proliferation and inducing T cell apoptosis [119] (Fig. 2). Interestingly, MGL has been demonstrated to bind STn antigen with a similar dead adhesion force than Tn [120, 121]. However, the impact of such interaction in immune responses is still poorly explored. Notwithstanding, it may, in part, contribute to STn associated immune tolerance [47, 122].

Siglecs are sialic acid-binding immunoglobulin-type lectins widely expressed in immune cells. These receptors selectively recognize α[2, 3], α[2–6] or α[2–8] sialic acids on the cell surface of cancer cells [123], including the broadly studied STn, ST antigens and sialylated N-glycans (Fig. 2). These Ig-type lectins are subdivided into CD33-related siglecs (-3, 5, 6, 7, 8, 9, 10, 11 and 14), showing high degree of sequence homology, and Siglec-1 (Sialoadhesin, CD169), Siglec-2 (CD22), Siglec-4 (MAG) and Siglec-15, which show low sequence similarity [123]. Siglec-2 and most CD33-related siglecs (except Siglec-14 and Siglec-15) have one or more cytosolic immunoreceptor tyrosine-based inhibitory motifs (ITIMs), actively supressing signals coming from receptors associated with immunoreceptor tyrosine-based activation motifs (ITAMs) [124]. In this context, siglecs are able to modulate tolerogenic functions in DCs, preventing expansion of effector CD4( +) and CD8( +) T cells and increasing Treg-cell numbers [125]. In particular, STn and ST antigens seem to be potent agonists of inhibitory siglecs, as macrophage Siglec-15 [48] and -9 [126], respectively, inducing tumour-associated macrophage (TAM)-like phenotypes and permitting immune scape (Fig. 2). Altogether, these glycan binding receptors interactions with tumour cell aberrant glycosylation constitute a novel class of immune checkpoints, offering potential for clinical intervention, including APC targeted glycovaccines envisaging T cell anti-tumour responses [127, 128].

Interestingly, immune cells also change their glycophenotype upon maturation and activation [90, 129]. For instance, mature DCs upregulate glycosyltransferases involved in the expression of LacNAc, core 1 and sialylated structures, while downregulating genes involved in the synthesis of core 2 *O*-glycans [130]. On the same note, murine activated CD4 and CD8 T cells experience a significant reduction in sialylated biantennary *N*-glycans with terminal NeuGcα[2–6]Gal, while overexpressing Galα[1–3]Gal terminal sequences in response to glycosyltraferase *ST6Gal I* downregulation and *α1-3GalT* overexpression [131]. Moreover, TCR expression of *β*1-6GlcNAc *N*-glycans promotes Th2 cells polarization over Th1 responses [132]. Since tumour cells also express GBRs with immunomodulatory potential that can bind these glycoepitopes, these findings are expected to also have implications in tumour cell recognition and tumour clearance responses [133]. In a very clear example, the glycophenotypes of T helper cells can directly modulate their susceptibility to Gal-1, which is frequently secreted by tumour cells [133, 134]. Namely, while Th1- and Th17-differentiated cells display a repertoire of cell surface glycans critical for Gal-1-induced cell death, Th2 cells present a different set of cell surface sialoglycoproteins that protects them from Gal-1 binding, thereby preventing inflammatory responses [135] (Fig. 2). In line with these observations, blockage of tumour Gal-1 interactions with activated T cells has been demonstrated to potentiate effective immune responses against tumour cells [134].

In summary, the crosstalk between GBRs and glycans on tumour and immune cells frequently leads to poor tumour specific responses. This is mainly driven by immune cell tolerogenecity, increased immune cell death and arrested proliferation, as well as promotion of non-effector T cell phenotypes as Tregs and Thelper cells. These findings highlight mechanistic interactions that are suitable for therapeutic intervention, which could be of major importance for immune checkpoint inhibitors non-responders that are currently faced with very limited therapeutic options.

## Models for cancer glycobiology and glycoimmunology

### Models for glycobiology research

**Fig. 3:**
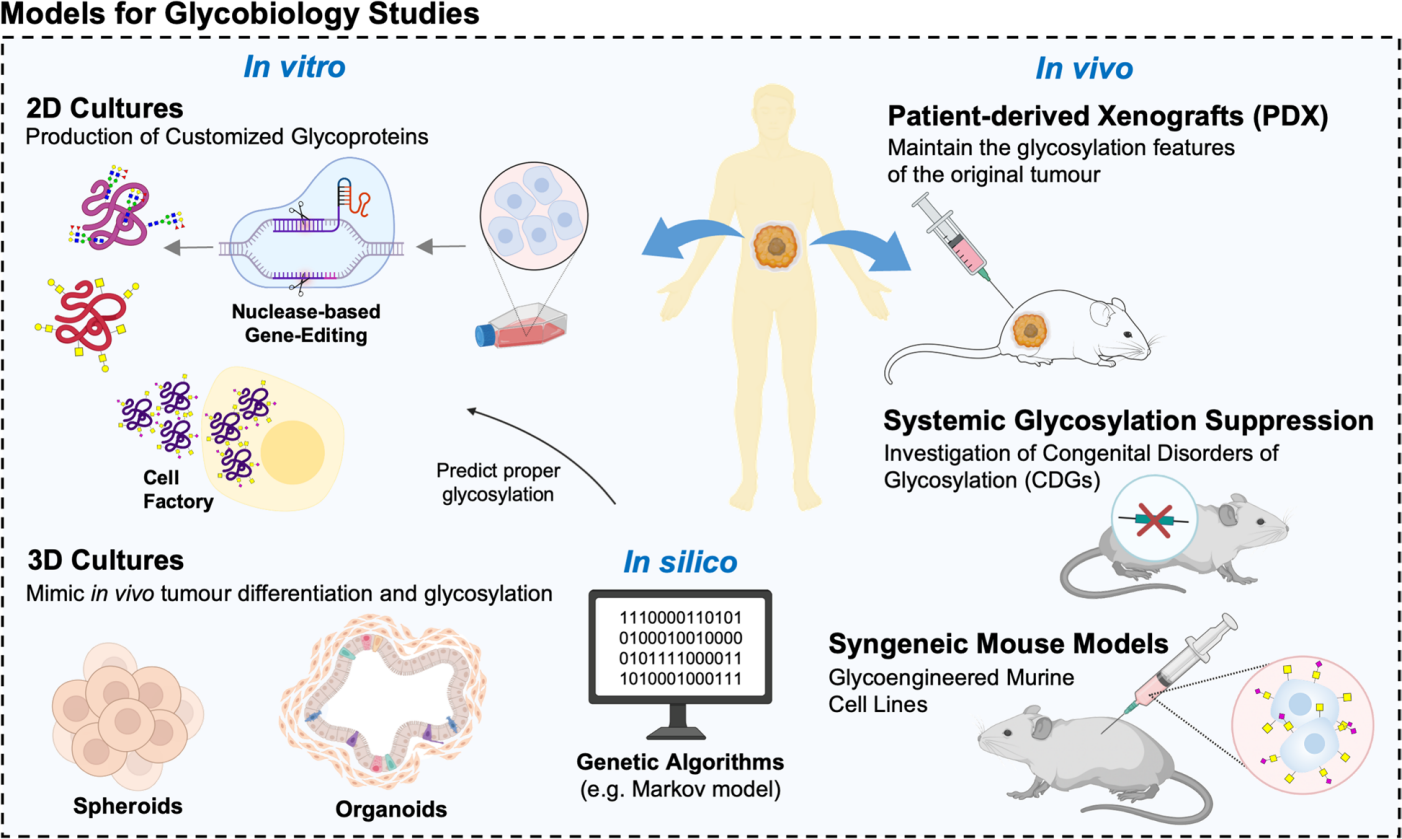
Current research tools and models for glycobiology studies. Cancer cell models have been extensively explored as primary research tools. Advances in gene editing technologies have enabled the precise and stable modulation of glycan biosynthesis pathways in mammalian cells, allowing the interrogation of glycosylation functional impact and the production of therapeutic glycoproteins. Cell factory technologies have been used for glycovaccines production, surpassing glycoproteins scalability challenges. The focus is now on progressing beyond 2D genetic glycoengineering towards 3D organotypic models, including high-throughput 3D spheroid cultures employing cell lines without or without genetic manipulation. Notwithstanding, the costly and time-consuming nature of these models remain major challenges. The introduction of computationally supported methods for glycoengineering constitutes the next logical cornerstone to address these limitations. Several transgenic mouse models reflecting the systemic impact of glycan deficiencies have also been developed and extensively employed as pre-clinical tools. Humanized models as patient-derived cancer xenografts (PDX) may add additional translational value. However, the loss of the immune system component poses as a major limitation. Alternatively, the adoption of syngeneic animal models developed from allografted glycoengineered cell lines, which can retain intact immune systems and provide the necessary means to address impact of glycosylation, could pose a valuable tool

Cancer glycobiology and glycoimmunology have extensively explored cancer cell models has primary research tools, building on its easy manipulation under co-culture with immune cells in controlled in vitro settings. Notably, most relevant cancer-associated glycome alterations, *e.g.* the overexpression of immature *O*-GalNAc glycans, are the result of poorly understood and, therefore, difficult to reproduce microenvironment cues. Advances in gene editing technologies have enabled the precise and stable modulation of glycan biosynthesis pathways in mammalian cells, inducing homogeneous cancer glycophenotypes for exploring glycosylation biological functions [136, 137]. Currently, mammalian cells glycoengineering relies mostly on nuclease-based gene-editing methods, including CRISPR-Cas9 technology [136, 138, 139]. The generalization of these approaches has been greatly facilitated by the pioneer work at Wandall’s lab [136, 138, 140, 141], which delivered a validated gRNA library for CRISPR/Cas9 targeting of the human glycosyltransferase genome [142]. This technology has been used to generate a wide number of different cancer cells expressing immature *O*-GalNAc glycosylation [143–145] which, to great extent, support our current knowledge on their role in heath and disease [146]. However, cellular glycoengineering for modulation of protein *N*- and *O*-glycosylation is being explored beyond its functional dimension, constituting an important tool for producing therapeutic glycoproteins [147, 148] (Fig. 3). Cell factory technologies have been used for glycovaccines production [149], surpassing low yields, scalability challenges and costs of glycopeptides synthesis using chemical approaches [128, 150] (Fig. 3). Currently, several pre-clinical glycovaccines for cancer and infectious diseases rely on these models as primary sources of antigens [149, 151, 152].

The focus is now on progressing beyond 2D genetic glycoengineering towards 3D organotypic models [153] (Fig. 3). As a result, the goal of establishing tissue libraries with altered glycosylation for therapy testing as well as cell differentiation, morphogenesis, cell–cell interactions, and cell–matrix interactions studies is currently at close range. However, the time consuming and costly nature of such models associated with the need for cell immortalization for long term studies, poses as a major operational limitation. Interestingly, high-throughput 3D spheroid cultures employing cell lines without genetic manipulation have been found to better mimic in vivo tumour differentiation and glycosylation features when compared to their 2D counterparts [154] (Fig. 3). However, a comprehensive characterization of the glycome, ideally supported by single-cell resolution, should be undertaken to fully disclose the potential of these approaches. Nevertheless, the new generation of 3D cellular models has already demonstrated potential for glycosylation studies [155] and may be of great interest for glycan-based therapy development. Notwithstanding, the costly and time-consuming nature of these models associated with challenges reproducing the complex and heterogeneous nature of the human glycome remain major challenges. The introduction of computationally supported methods for glycoengineering may constitute the next logical cornerstone to address these limitations. Genetic algorithms, as the Markov model, may be used to identify cell lines and clones requiring minimal intervention to achieve the desired glycophenotypes (Fig. 3). These strategies may also help defining operational parameters (*e.g.* amount of starting material; microenvironmental cues) towards the best possible predictable outcome [156, 157]. Ultimately, these approaches also offer a flexible and user-friendly platform to optimize mammalian cell factories production, optimizing biopharmaceutical production efforts.

Less explored, but far more informative approaches imply the use of cancer animal models derived from different approaches, *e.g.* glycogene glycoengineering, grafting of cell models and tumours, or induction of lesions by either chemical of physical methods. Several transgenic mouse models reflecting the systemic impact of glycan deficiencies have also been developed and extensively employed as pre-clinical tools. The most frequently used mouse models display induced deficiency of core 1-derived *O*-glycans [158, 159], branched *N*-glycans [160], *O*-GlcNAcylation [161], and multiple enzymes determining congenital disorders of glycosylation (CDGs) [162]. These models have been used for investigating CDGs progression and associated therapeutic options [163], susceptibility to intestinal inflammation [160], gut microbial ecology [164], host physiology [165], T cell development [166, 167] and cancer progression [168] (Fig. 3). However, the net abrogation of a glycophenotype in an animal may not fully reflect the context-dependent nature exhibited by subpopulations of cells in tumours. As such, humanized models may add additional translational value to current models. For instance, patient-derived cancer xenografts (PDX) were demonstrated to preserve many of the original tumour molecular features, including glycosylation signatures, providing a suitable model for therapeutics testing [169], while being a relevant tool for soluble glycobiomarker discovery [170] (Fig. 3). However, the loss of the immune system component poses as a major limitation. Alternatively, the adoption of syngeneic animal models developed from allografted glycoengineered cell lines, which retain intact immune systems and provide the necessary means to address impact of glycosylation [171, 172], could pose a valuable tool. The induction of tumours in animal models also poses as a valuable alternative for glycobiology, as we have previously demonstrated building on chemically induced bladder tumours [173]. However, these solutions are yet to be generalized and require careful consideration of intrinsic molecular and immune system differences between humans and animal models in experimental design.

### Models for cancer glycoimmunology

The current cancer glycoimmunology state-of-the-art has mainly focused on the immune modulation promoted by cancer-associated glycans, emphasizing the study of glycans as novel immune checkpoints. This quest has been mostly backed by glycobiology models, including molecular simulation methods of docking and protein interactions [96], where glycan and glycan binding receptor interactions probabilities are modulated. Furthermore, glycoengineered cell lines co-culture with immune cells, immunohistochemistry validations in tumour samples, and mouse models have also aided the discovery of immunosuppressive interactions [174]. Notwithstanding, in vitro methods frequently lack the necessary tumour microenvironment context, and mouse models have been filling this gap. However, fundamental physiological differences between mouse and human immune receptors and glycosyltransferases expression could be hampering clinical translation and ICI-based immunotherapy development. For instance, mice only have 9 functional homologues of the 15 human siglecs, with mouse siglec 3 also lacking the ITIM motif found in humans [175]. Furthermore, most research on DC-SIGN has relied on in vitro studies, since there are eight genetic homologs of human DC-SIGN in mice with no clear DC-SIGN ortholog [176]. Accordingly, the physiological role of this receptor in vivo has been hard to address. Also, the phylogeny of glycosyltransferase genes influences the endogenous expression of several ligands of these receptors in mice, with some enzymes being absent from the mouse genome as *FUT3*, *FUT5* and *FUT6* that support the expression of lewis antigens in humans [177]. Bioimaging, enzymatic synthesis of relevant glycopeptides [117] and glycoengineered cell lines protein production have been supporting studies of glycopeptide antigenicity and analysis of antibody responses in immunocompetent mice, as well as the induction of specific T cell hybridomas to investigate APC functions [16].

Currently, the field is progressing to organotypic and humanized scale. An elegant example employs patient derived cancer organoids (PDOs) expressing cancer specific Carcino-Embryonic Antigen (CEA) glycoconjugates, which allowed the refinement of immunotherapies based on bispecific antibodies targeting CEA on cancer cells and CD3 on T cells [178]. Glycoengineered transgenic mice have also been extensively used in this context, despite posing limitations regarding incapacity to mimic the context dependent nature of glycosylation. For instance, a human ex vivo pre-clinical study using mucosal T lymphocytes from patients with ulcerative colitis (UC) has highlighted a possible targeted-specific immunomodulatory role for *N*-acetylglucosamine (GlcNAc) metabolic supplementation [179]. Using transgenic mouse models missing branched *N*-glycosylation potential (*Mgat5 − / −)*, Dias et al*.* has demonstrated that GlcNAc supplementation has the potential to enhance T cell receptor branched *N*-glycosylation, thereby controlling T cell-mediated immune responses at the intestinal mucosa and reducing UC severity and progression [179]. Ultimately, this study proposes a simple rescue therapy approach for patients with UC, with potential to avoid unnecessary toxic effects of mainstay treatments. Using human cancer cell lines and the above mentioned (*Mgat5 − / −)* mouse model, the immunoediting capacity of complex branched *N*-glycans and the impact of the removal of such epitopes in cancer cell immunogenicity was also investigated [73]. In brief, the removal of branched *N*-glycans exposed immunogenic mannose antennas that potentiated immune recognition by DC-SIGN-expressing immune cells, resulting in an effective antitumour immune response [73]. These findings highlight the therapeutic efficacy of glycosylation modulation as a strategy to potentiate immune recognition. On the same note, the (*Mgat5 − / −)* mouse model was also explored in the context of autoimmunity and the negative impact of branched *N*-glycans in T-cell activation was reenforced [180]. However, a second mechanism of immunoediting was suggested. Namely, deficiency in *Mgat5* was determined to lower T-cell activation thresholds by directly enhancing TCR clustering and potentiating T cell activation [180].

Overall, glycoimmunology is still at its infancy in terms of exploring the full potential of humanized models of the immune system, relying heavily on 2D in vitro co-culture of immune and cancer cells and glycoengineered mice. Of note, transgenic mice presenting glycosylation deficiencies display persistent comorbidities. For instance, Mgat5-deficient mice consistently show kidney autoimmune disease, enhanced delayed-type hypersensitivity, increased susceptibility to experimental autoimmune encephalomyelitis, and reduced depression‐like phenotype [180, 181], suggesting not only autoimmune disease predisposition but also expected behavioural changes. This constitutes a major limitation that should be accounted for facing clinical translation. Given these insights, progress from co-culture systems towards 3D humanized and organotypic settings is expected to pose as a mainstay approach in the future. However, the field could also extensively benefit from the outstanding advances in immune system models and machine learning algorithms already explored by immunologists.

## Emerging models for translational immunology: opportunities for glycoimmunology

### Murine models of the human immune system

**Fig. 4:**
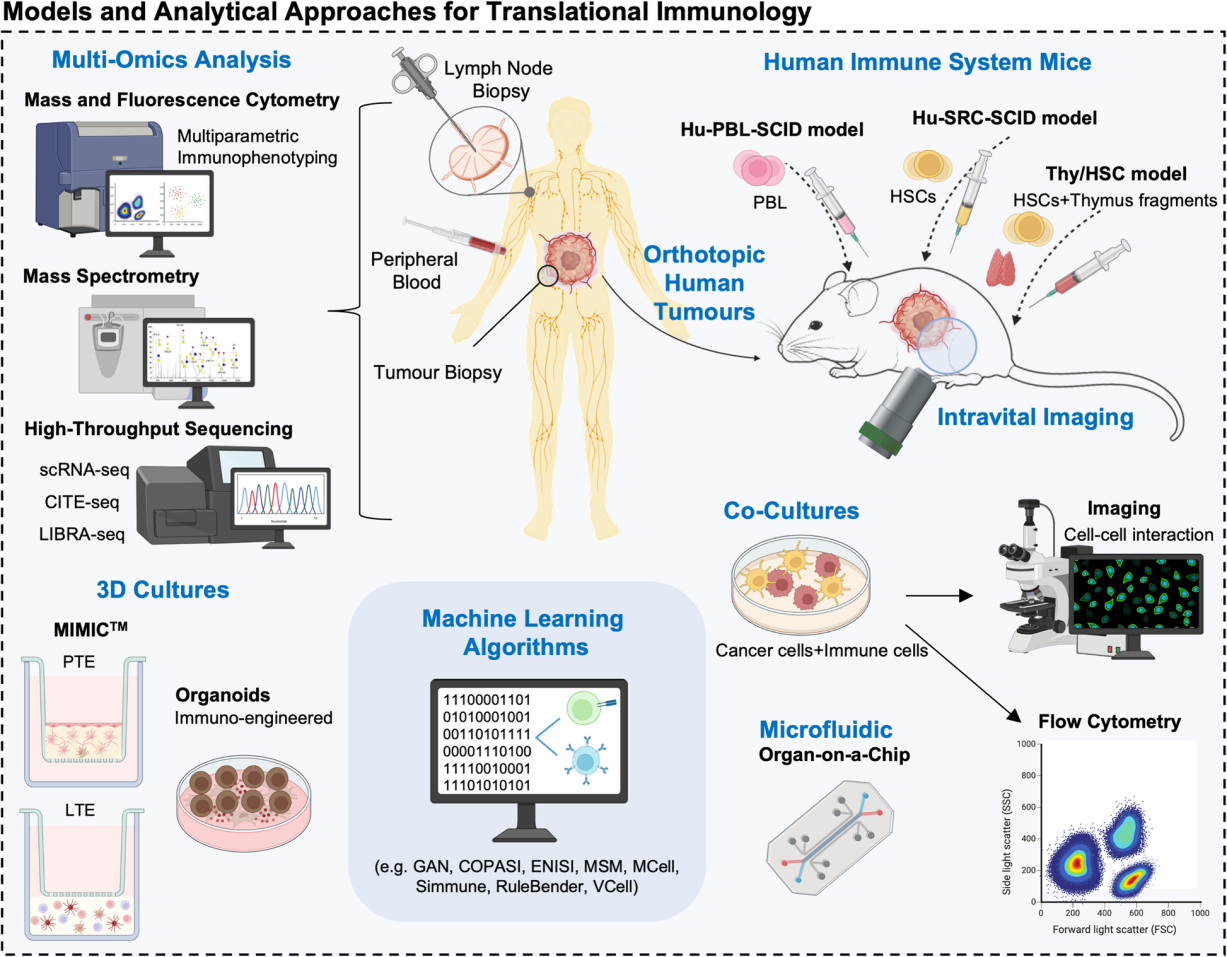
Emerging models for translational immunology. Humanized murine models have been a step forward in mimicking human immune traits. Some of the more broadly used humanized models include the Hu-PBL (peripheral blood leukocyte)-SCID model, SRC (SCID repopulating cell)-Hu model, and the Thy/HSC model. In these examples, immunodeficient mice can be engrafted with functional human cells and tissues, including orthotopic human tumours and human hematopoietic stem cells (HSC) that develop into functional human immune systems. Mass and fluorescence cytometry of peripheral blood samples have been key technologies explored for multiparametric human immunophenotyping in oncology settings, revealing interindividual variations and tissue specialization of immune subsets. In parallel, high-throughput sequencing has enabled the unlocking of the extraordinary heterogeneity of the immune repertoire from a single sample of blood or tissues. In this context, sophisticated machine learning algorithms are being used to integrate large sequencing datasets. Regarding in vitro models of the human immune system, some challenging approaches have been proposed, including human modular immune in vitro constructs (MIMIC™) and organoid-like cultures. The MIMIC system technology is a 3D structure composed by a peripheral tissue equivalent (PTE) and a lymphoid tissue equivalent (LTE), allowing multivariate studies. Immuno-engineered organoids have also been used as an alternative to overcome the limitations associated with animal models, 2D systems, and previously existing 3D models

There is growing awareness regarding the need to progress towards human-based systems able to support the translation of findings from fundamental immunology studies to the clinics. We will first focus on novel murine models of the human immune system, given their preferred use in onco-immunology (Fig. 4). In fact, most of our basic understanding on the subject has been clarified by these models, given their manageability and readily available reagents and tools. However, the incapacity of mice to mirror human immune responses poses as a great limitation for clinical translational studies [182–187]. This is mainly due to interspecies differences between cytokines and cytokine receptors, incapacity to reflect immune senescence, lack of genetic heterogeneity, inability of many common pathogens to develop in mice, among others [188–192]. Notwithstanding, humanized murine models have been a step forward in mimicking human immune traits. Some of the more broadly used humanized models include the Hu-PBL (peripheral blood leukocyte)-SCID model, SRC (SCID repopulating cell)-Hu model, and the Thy/HSC model, each having its own advantages and disadvantages reviewed elsewhere [193] (Fig. 4). In these examples, immunodeficient mice can be engrafted with functional human cells and tissues, including orthotopic human tumours and human hematopoietic stem cells (HSC) that develop into functional human immune systems [194–196]. These models rely on tissue engineering approaches to create a humanized microenvironment, as opposed to simply engrafting cells [197]. Nevertheless, there are substantial costs underlying the development of humanized models, a restrictive supply of animals, and ethical concerns related with animal model research. Moreover, several organs are unable to interact with the grafted human leukocytes due to species specificity, potentiating conflicting results. Furthermore, transplantation is performed using identical batches of stem cells in highly inbred animals, which fails to reflect the necessary human heterogeneity [198]. To address these hurdles, co-engraftment of several human tissues with human hematopoietic stem cells has been proposed to improve on humanization of immune responses [199]. However, these tools will never be fully humanized, urging the constant development and refining of new models.

### Biopsy-based models

Addressing these difficulties, several studies sought to investigate immune cells from peripheral blood and tissue biopsies of tonsils and lymph nodes (LN) of human patients. Mass and fluorescence cytometry of peripheral blood samples have been key technologies explored for multiparametric human immunophenotyping in oncology settings, revealing interindividual variations and tissue specialization of immune subsets [200–203] (Fig. 4). In parallel, high-throughput sequencing has enabled the unlocking of the extraordinary heterogeneity of the immune repertoire from a single sample of blood or tissue [204]. In this context, sophisticated machine learning algorithms are being developed to integrate large sequencing datasets, infer on T-cell specificity and B cell receptors heterogeneity as well as vaccine-specific antibody production, which could provide key knowledge regarding adaptive immune responses [204–208]. On the same note, repertoire analysis of antigen specific TCRs has been suggested as an important readout to assess vaccination’s ability to generate memory cells and the necessary clonal expansion for immune protection, reinforcing the immediate clinical translation of peripheral blood analysis [209]. Furthermore, RNA-seq, ChIP-seq, and ATAC-seq have been providing key information on immune genes regulome, offering insights on epigenomic state and functional aspects of human immune cell types [210–214]. Also, single-cell omics approaches, including highly multiplexed simultaneous detection of RNAs, proteins, miRNAs, and gene-specific mRNA, has been elucidating the identity and functional state of critical immune subsets, while opening new avenues for the characterization of cellular metabolism [215–218]. More importantly, the comprehensive integration of multi omics information has brought us one step closer to identifying global immune signatures associated with clinical outcome, even when patients’ cohorts are small and heterogeneous [219] (Fig. 4). Ultimately, this high-resolution systems-level immune monitoring offers a roadmap for the development and evaluation of immunotherapies.

In turn, LN biopsies have also been employed in human immune repertoire studies as well as vaccine and immune checkpoint inhibitors (ICI) response research. Biopsies of vaccine-draining lymph nodes have allowed the in vitro generation of the same antibodies observed in the blood of vaccinated patients [220], which has been facilitating investigation on human antibody activity and therapeutic antibodies development. Furthermore, high-dimensional mass cytometry of sentinel LNs biopsy samples combined with T cell receptor repertoire sequencing has allowed the interrogation of T follicular helper (Tfh) cells in primary human LNs [221], which is expected to aid in the therapeutic manipulation of cellular functional capacity to improve antibody responses to vaccination [222]. Most of these cellular processes are practically indetectable in peripheral blood, as such, gathering information from fine needle aspirates of human LN, including tumour draining LN, has emerged as a valuable tool for human immunization studies [220, 223–225]. In parallel, regional LN immune profiles have been suggested as predictive biomarkers for immune checkpoint inhibitor response, challenging the view that ICI activity occurs primarily at the tumour site [226] and focusing attention on tumour draining lymph nodes [227].

### In vitro* and lab-on-a-chip models for validation studies*

Even though blood and liquid biopsy-based assays have been extensively informative, validation studies have been mainly undertaken building on studies in vitro. Co-cultures between cancer cells with immune cells isolated from blood or immune tissue fractions (human tonsils and lymph nodes) sorted cells are by far the preferred strategies [228–230] (Fig. 4). In this context, antigen presenting cells, as DCs and macrophages, interactions with tumour cells and downstream immune effectors, as T cells, can be closely monitored. By exploring lymphocyte–APC contacts, many signalling molecules, including receptors, enzymes, adaptors, and secondary messengers, have been identified and support the current state of the art [231–234]. Furthermore, imaging of cell–cell contacts has led to an appreciation of the remarkable supramolecular changes, covering molecular structure, conformational changes, mobility of bound species, intracellular activity of proteins, intracellular localization, aggregation state of receptors, mobility at the plasma membrane, and cell morphology [235]. Even though intravital imaging of murine models has been extensively used towards this end, the physiological context may have to be compromised to obtain higher resolution. Given these obstacles, a combination of low-resolution in vivo imaging and high-resolution in vitro experiments could allow advances in the study of APCs engagement, lymphocyte activation and tumour cell elimination or evasion processes.

Regarding in vitro models of the human immune system, some challenging approaches have been proposed, including human modular immune in vitro constructs (MIMIC™) and organoid-like cultures. The MIMIC system technology is a 3D structure composed by a peripheral tissue equivalent (PTE) and a lymphoid tissue equivalent (LTE) [236] (Fig. 4). The PTE is comprised by a monolayer of human umbilical vein endothelial cells (HUVEC) cultured above a 3D extra-cellular collagen matrix, upon which patient peripheral blood mononuclear cells (PBMCs) are seeded. This module can simulate innate immune responses and gives rise to four main mononuclear populations. Namely, immature CD14 + DCs precursors, immature CD14– DCs, and mature DCs. A fourth population is more macrophage-like and is retained in the matrix. The LTE is essentially an artificial lymph node, simulating the adaptive immune response. Dendritic cells, follicular dendritic cells, T- and B-cells are applied in sequential order to mimic the immune response expected in vivo. As such, DC–T-cell interactions, antigen–B-cell interactions, T-cell and B-cell interactions, Th1 or Th2 polarisation bias, antigen-specific antibody production, and cytotoxic T-cells activity, can all be assessed from this in vitro module [226, 237]. In turn, immuno-engineered organoids have been used as an alternative to overcome the limitations associated with animal models, 2D systems, and previously existing 3D models (Fig. 4). Namely, organoids simulate the physiological organ structure, can include important stromal components, can be specifically genetically engineered, do not encompass ethical conflicts, and allow different degrees of malignancy to be cultured. Moreover, patient derived organoids allow tumour and immune cells to be cocultured, which is critical for immuno-oncology studies and immunotherapeutic screening [238–240]. For instance, immuno-engineered organoids have been used to accelerate the induction of a germinal centre (GC)-like phenotype in B cells to support a controllable immunoglobulin class-switching reaction [241], surpassing all previously described 2D ex vivo systems for GC-like phenotypes. Furthermore, murine- and patient-derived organotypic tumour spheroids retaining autologous lymphoid and myeloid cell populations have been used to anticipate response to cancer immunotherapy, including antibody-based immunotherapy and ICI, oncolytic virus therapy, and adoptive cell transfer therapy [242, 243]. Accordingly, complex immune–organoid cultures have been providing real time tools for pre-clinical testing of therapeutic combinations and facilitating precision immuno-oncology efforts [244, 245]. Interestingly, immuno-engineered organoids research has been hand-to-hand with dynamic microfluidics and organ-on-a-chip solutions for oncoimmunology [246, 247]. Accordingly, these microfluidics multicellular systems have been employed in the study of soluble immune checkpoint inhibitors [248, 249], sequencing of antibodies secreted during innate immune responses [250], the crosstalk between cancer and immune cells [251], immune cell migration [252, 253], the cytotoxic activity of TCR-engineered T cells [247], and the impact of anti-tumour chemotherapy/ radiation and combination therapies on tumour and immune cells [254–256]. Altogether, an encouraging body of literature has demonstrated that OncoImmuno multicellular chips provide a flexible and valuable alternative to animal models, mostly due to their affordability and accuracy in drug testing screenings while recapitulating the relationships between the immune system and cancerous tissues [246].

## High-throughput single cell technologies for translational immunology: opportunities for glycoimmunology

There has been an increasing awareness that solid tumours are not discrete bodies but an interconnected network of cell populations, which can drive tumour progression and therapy resistance in a clonal dependent manner [257, 258]. However, traditional molecular profiling techniques have focused on the tumour bulk disregarding discrete contributions from relevant subpopulations. In fact, cancer cells in a tumour display distinct cellular morphologies, gene expression patterns, proliferation rates, metastatic potential and, sensitivity to treatment, which poses a major obstacle to understanding and treating cancer [257, 258]. Accordingly, the pursue for single-cell resolution tools has opened new avenues for single-cell characterization, including genomics [258], transcriptomics [259], epigenomics [260], proteomics [261] and metabolomics [262] sequencing. This has been mostly aiding therapy resistant clones’ phenotyping and immunotyping of immune cells.

Currently, several high-throughput and high resolution techniques are becoming standard practice in deciphering tumour heterogeneity in a multiplexed manner. For instance, cytometry by time of flight (CyTOF) allows non-targeted and comprehensive cellular characterization, offering a broader vision on cell signalling pathways at various cell stages [263]. Cell staining in CyTOF protocols are fairly similar to standard flow cytometry procedures, taking advantage of antibodies labelled with stable metal isotopes and culminating in charged plasma vaporization of cells prior to TOF unit analysis [264]. CyTOF offers several advantages to traditional flow cytometry, including highly multiplexed target detection, allowing the scale up to more than 40 intracellular and extracellular targets in a single run [265], while surpassing fluorescence compensation issues. However, mass compensation and oxidative processes need to be considered, similarly to other widespread mass spectrometry techniques [266]. On the same note, full spectrum flow cytometry (SFC) allows the same multiplexed analysis at a single cell level than CyTOF, with similar data readouts [267]. However, SFC offers the possibility of using all conventional flow cytometry reagents, from FACS buffers to fluorophore-tagged antibodies, further driving the adoption of SFC as a mainstay of single-cell phenotyping. Briefly, full spectrum flow cytometers detect the entire spectral signature of a fluorophore through various tandem detectors, allowing the multiplexed acquisition of more than 30 cellular targets at a time, even when using highly overlapping fluorophores [268]. Moreover, full spectrum flow cytometers are able to use autofluorescence values to improve data quality, surpassing conventional flow cytometers both in resolution as well as in compensation issues [269]. Notwithstanding, the search for automated and extremely multiplexed approaches has led to the development of cellular indexing of transcriptomes and epitopes by sequencing (CITE-seq), which brings together surface protein phenotyping and single-cell RNA sequencing (scRNA-seq) [270]. This technique takes advantage of antibodies conjugated to oligonucleotides, namely a unique antibody oligonucleotide barcode that identifies the antibody/marker and a terminal poly(A) sequence. When cells stained by these antibodies are lysed, the marker poly(A) tail is captured by hybridizing with beads covered in poly(dT) oligonucleotides, following scRNA-seq [271]. Since CITE-seq relies on oligonucleotides instead of fluorophores and has sequencing as a readout, there is no known limit for the number of simultaneous markers that can be analysed on a single run, allowing a scale up to more than 200 multiplexed indicators. Accordingly, CITE-seq allows measurement of a potentially unlimited number of protein markers in parallel to transcriptomes, enables sample multiplexing, robust multiplet detection and super-loading of scRNA-seq platforms. Furthermore, some high-throughput technologies have been developed to address specific questions. For instance, the need to pair B cell receptor sequence to antigen specificity at a single-cell level is currently addressed through LIBRA-seq [272]. LIBRA-seq (linking B cell receptor to antigen specificity through sequencing) enables high-throughput mapping of paired heavy- and light-chain BCR sequences to their cognate antigen specificities. B cells are mixed with a panel of oligonucleotides conjugated to recombinant antigens so that both the antigen and BCR sequence are recovered during paired-chain BCR sequencing experiments and bioinformatically mapped to single cells [272]. Ultimately, LIBRA-seq enables mapping of monoclonal antibody sequences, theoretically unlimited in number, and facilitates rapid identification of cross-reactive antibodies, being an integral tool for antibody discovery as well as vaccine and immunotherapy development.

In addition to the above-mentioned technologies, advances in multilayer microscopy have been aiding the accurate assessment of tissue architectures and microenvironments, while allowing single cell resolution. In this context, co-detection by indexing (CODEX) multiplexed immunofluorescence was created to address the need for multi-marker spatial analysis. This need derives from the notion that not only the composition of tissues but also the spatial proximity of cell subtypes affects biological outcomes. CODEX can be applied to human and mouse fresh frozen or FFPE tissues, which are incubated with a cocktail of oligonucleotide tagged primary antibodies without fluorophores. During the automated processing of tissues in the CODEX platform, dye-labelled oligonucleotides (CODEX reporters) bind to their complementary antibodies and give out fluorescence signals, following the successive removal and addition of new reporters for up to 16 cycles. This can generate spatial information for up to 46 protein biomarkers [273]. As such, CODEX technology automates whole tissue imaging, allowing complex single cell phenotyping and discovery of novel phenotypes, while disclosing spatial interactions within tissues [274]. In a similar way, spatial transcriptomics has also been extensively pursued through NanoString technology, enabling researchers to locate transcripts down to the subcellular level and providing an unbiased map of RNA targets throughout tissue sections. Herein, target RNA is directly tagged with specific capture and reporter probes, creating unique target-probe complexes for each target. These complexes are automatically immobilized in an imaging surface and the sample is scanned by an automated fluorescence microscope, enabling digital quantitation of hundreds of unique targets in a single reaction.

Adding to the advances brought by CyTOF, mass spectrometry (MS) has reached the technological readiness to enable more detailed unbiased proteome characterization close to single cell resolution [275–279]. Opportunities, limitations, and key enabling milestones towards clinical translation regarding this technology have been recently revised [280–283] and therefore, will not be discussed in detail in this review. However, improvements in sample processing, separation and MS instrumentation have made possible to quantify over 1000 proteins from individual mammalian cells, which until very recently could only be achieved with an input of thousands of cells. The challenge is now set on expanding the dynamic range of identified proteins, also enabling the identification of low abundance proteins and post-translational modifications, including glycosylation. In parallel, there have been significant advances in terms of spatially resolved proteomics at single cell resolution [284–287]. Nevertheless, single cell glycomics and glycoproteomics remains a challenging enterprise that has just began to be tackled. Namely, imaging mass spectrometry is currently being employed for glycomic characterization of tumour sections, allowing identification of glycome signatures according to tissue distribution and underlying associations with relevant histopathological features [288–290]. Furthermore, innovative workflows such as the SUrface-protein Glycan and RNA-seq (SUGAR-seq) allowed the combo analysis of glycans, extracellular epitopes, and the transcriptome at the single-cell level, progressing over existing technologies in terms of mapping cellular transcriptional and phenotypic features [291]. Integrated SUGAR-seq and glycoproteome analysis has led to the characterization of tumour-infiltrating T cells glycophenotypes, mirroring their epigenetic and functional state and holding potential for advances in cancer glycoimmunology. Very recently, we have also materialized the concept of glycoproteogenomics, which builds on comprehensive integration of RNAseq-data to customized databases used in protein annotation [89, 292]. This has allowed deeper access to the glycoproteome, now requiring translation to single cell analysis. Collectively, the combination of genomics, transcriptomics, and proteomics in the context of single cell analysis will provide tremendous advances in terms of understanding how gene products interact to produce a cellular phenotype. The integration of glycomics and glycoproteomics will also be key to identify protein functional targets. The exploitation of these concepts and technologies in glycoimmunology will allow to gain knowledge on the role played by glycans in the crosstalk between cancer cells and the immune system and the relevance of the immune cells glycome in health and disease.

## Machine learning tools for immunological modelling

Finally, all the disruptive technologies mentioned above provide high resolution molecular and cellular insights, which require potent machine learning tools to integrate information and accelerate the translation of these insights into therapies. Accordingly, several user-friendly computational tools that facilitate immunological modelling have been developed, ultimately providing an additional quality-control mechanism that improves the rigor and reproducibility of immune studies [293]. For instance, deep-learning Generative Adversarial Networks (GAN) can predict how an immune cell migratory path will evolve based on a time lapse microscopy video file [294]. In oncoimmunology settings, GAN provides a meaningful estimation of the probability for each immune cell to physically interact with cancer cells in their vicinity. Moreover, this long-term prediction of cell trajectories may reduce the spatial–temporal burden of video sequences storage, solving a significant bottleneck of the experimental pipeline. On the same note, Complex Pathway Simulator (COPASI) [295], ENteric Immunity Simulator (ENISI) MultiScale Modeling (MSM) [296], Simmune [297], Monte Carlo Cell (MCell) [298], RuleBender [299], and Virtual Cell (VCell) [300] constitute several other comprehensive platforms for modelling that are used by immunologists. These tools provide cellular, spatial, and time-dependent simulations of immune processes [293], all of which holding promise for the systematic interrogation of complex pathways. Furthermore, bioinformatics tools can also be used to integrate publicly available curated data, taking advantage of pre-existing information to advance beyond the current state-of-the-art [301–307] in a way that often improves reproducibility by integrating data from multiple studies [308].

In summary, as we progress toward increasingly sophisticated humanized models of the immune system, several relevant stromal and microenvironmental cues driving malignancy have been identified. The spotlight is currently on the tremendous potential brought by multicomponent 3D models for personalised oncoimmunology. These approaches supported by multi-omics characterization at single cell resolution [309, 310] are positively contributing to better understand the role played by the cancer microenvironment in disease [311, 312]. The exploitation of bioinformatics and computational tools backed by artificial intelligence has been decisive towards this objective, setting a roadmap ready to be translated to cancer glycobiology.

## Concluding remarks

**Fig. 5:**
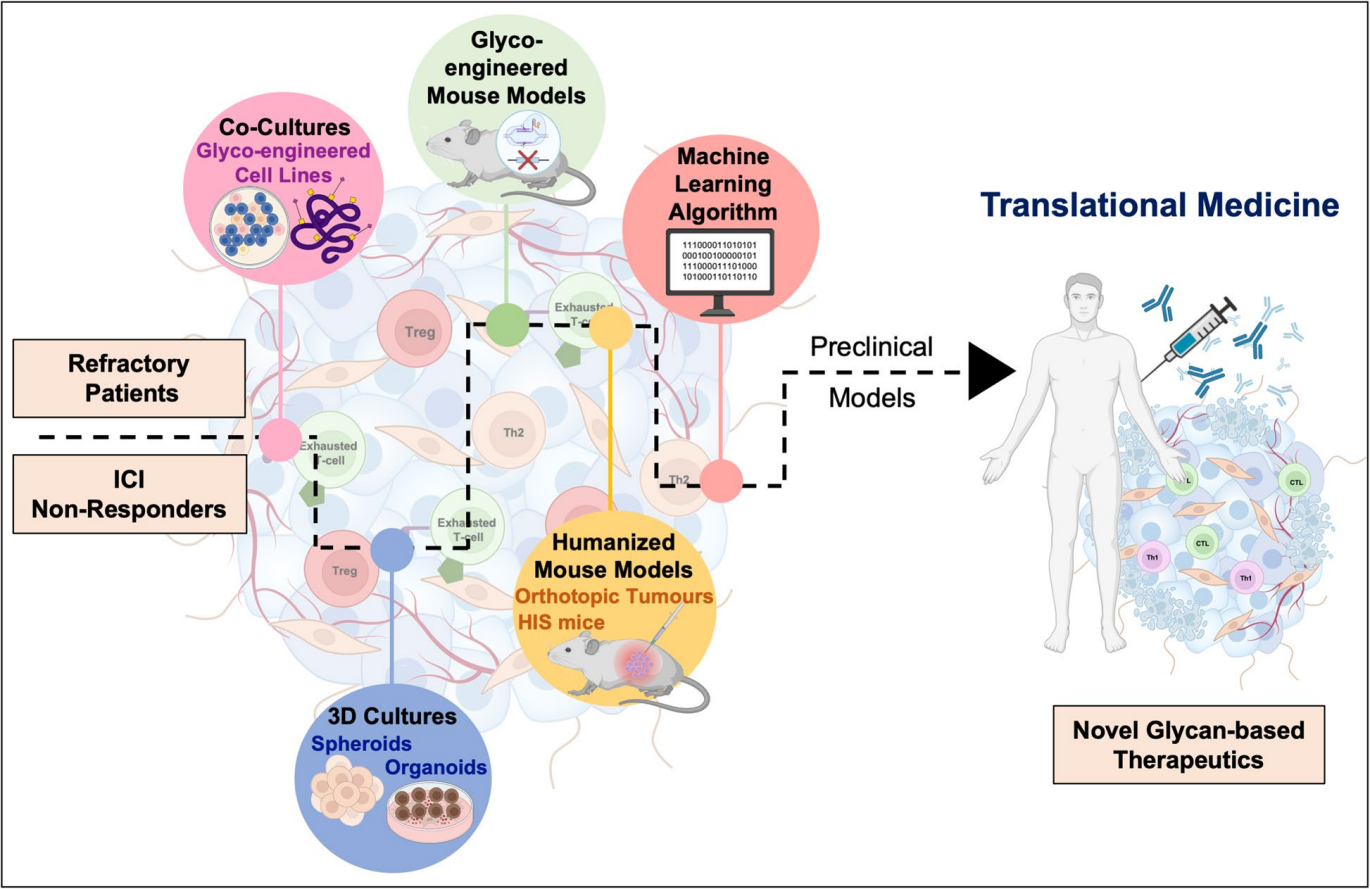
Roadmap towards novel glycan-based immunotherapy. Humanized in vitro and in vivo models are expected to hasten the translation of fundamental glycoimmunology research into glycan-based tools with theragnostic potential. Currently, the field heavily relies on short term in vitro co-cultures involving glycoengineered cell models and blood derived immune cells. Transgenic mouse models resulting from glycogenes editing have also been explored. The generalization of 3D systems, namely organoids, organs-on-a-chip, and humanized animal models will be of key importance to capture tumour heterogeneity and immune system contributions to tumour progression. The generalization of artificial intelligence approaches will also be decisive to integrate massive omics data into comprehensive models. We anticipate that such roadmap could speed the arrival of much needed novel therapeutic options, constituting valuable alternatives to current immunotherapies.

Immune checkpoint inhibitors against PD-1/PD-L1, or CTLA-4 have revolutionized the field of immunotherapy. However, immune suppression is supported by multifactorial intricate molecular networks, building on interplays between cancer and different types of immune cells, which we are only now beginning to understand. Furthermore, there has been an increasing awareness that discrete tumour cell populations can drive tumour progression and therapy resistance in a clonal dependent manner. Thereby, focusing on relevant subpopulations at the single cell level constitutes the next cornerstone in understanding and treating cancer. Multi-omics analysis at single cell resolution has greatly contributed to this objective, being decisive to bring the cancer glycome into the spotlight. As a result, there is growing awareness that glycans classically known to mediate cancer invasion and metastasis are also playing significant suppressive roles of both innate and adaptative immunity. As we continue to unveil the cancer glycome, it becomes pressing to invest on exploring the mechanistic aspects of glycan-GBR interactions towards the rational development of novel interventions. Notably, this knowledge has been successfully prototyped into blocking antibodies, glycoengineered vaccines and cellular immunotherapies, as CAR-T cells, with promising results in pre-clinical settings. However, few therapeutics ever get to surpass early clinical trial phases. We believe that the missing link between fundamental glycoimmunology knowledge and translational medicine may lay in the experimental models adopted by most studies. Currently, the field heavily relies on short term in vitro co-cultures, generally involving glycoengineered cell models and immune cells extracted from peripheral blood of healthy donors and cancer patients. To less extent, transgenic mouse models resulting from glycogenes editing have also been explored (Fig. 4). Even though these tools have successfully supported breakthrough advances in the field, they often lack the necessary humanized features and the underlying tumour microenvironment. Accordingly, the generalization of 3D systems, namely organoids, organs-on-a-chip, and humanized animal models, that are currently used in infectious disease, autoimmunity, oncology, and immunotherapy studies, will be of key importance for the glycobiology field. Finally, it is pressing to invest on the comprehensive molecular characterizations of cancer and immune cells by high-throughput genomics, transcriptomics, glycomics and (glyco)proteomics at single cell resolution. These new approaches will be key for dissecting tumour heterogeneity, including the precise characterization of immune cells constituting the microenvironment. This will translate into a deeper understanding about the role played by glycans and glycoconjugates in immune responses, cancer progression and dissemination, facilitating the identification of targetable molecules and the rationale design of novel and more effective therapies. The generalization of artificial intelligence approaches will also be decisive to integrate massive omics data, including glycomics and glycoproteomics, into comprehensive models (Fig. 5). We anticipate that such roadmap could speed the arrival of much needed novel therapeutic options, constituting valuable alternatives to current immunotherapies.

## Data Availability

Data sharing is not applicable to this article. All data analysed during the current study is available in the PubMed repository, https://pubmed.ncbi.nlm.nih.gov.

## Abbreviations

APC: Antigen presenting cells
ATAC-seq: Assay for transposase-accessible chromatin using sequencing
BCR B: Cell receptor
CAR-T: Chimeric antigen receptor T cell
CDGs: Congenital disorders of glycosylation
ChIP-seq: Chromatin immunoprecipitation sequencing
CITE-seq: Indexing of transcriptomes and epitopes by sequencing
CLR: C-type lectin receptor
CODEX: Co-detection by indexing
CRISPR: Clustered regularly interspaced short palindromic repeats
CTLA-4: Cytotoxic T lymphocyte-associated antigen 4
CyTOF: Cytometry by time of flight
ECM: Extracellular matrix
Gal: Galectin
GBR: Glycan-binding receptors
HSC: Human hematopoietic stem cells
hu-PBL: Human peripheral blood leukocytes model
ICI: Immune checkpoint inhibitor
IL: Interleukin
ITAM: Immunoreceptor tyrosine-based activation motif
ITIM: Immunoreceptor tyrosine-based inhibitory motif
LIBRA-seq: Linking B cell receptor to antigen specificity through sequencing
MHC: Major histocompatibility complex
MS: Mass spectrometry
PBMC: Peripheral blood mononuclear cells
PD-L1: Programmed cell death ligand 1
PDO: Patient derived cancer organoids
PDX: Patient-derived cancer xenograft
RNA-seq: RNA-sequencing
SCID: Severe combined immune deficiency
SFC: Full spectrum flow cytometry
SRC: Scid-repopulating cell
TAM: Tumour-associated macrophage
TCR: T-cell receptor
TIM3: T cell immunoglobulin and mucin domain 3
TME: Tumour microenvironment
